# Disposition of a Glucose Load into Hepatic Glycogen by Direct and Indirect Pathways in Juvenile Seabass and Seabream

**DOI:** 10.1038/s41598-017-19087-y

**Published:** 2018-01-11

**Authors:** João Rito, Ivan Viegas, Miguel A. Pardal, Isidoro Metón, Isabel V. Baanante, John G. Jones

**Affiliations:** 1 CNC - Center for Neuroscience and Cell Biology, Rua Larga, 1° Piso da FMUC, University of Coimbra, 3004-504 Coimbra, Portugal; 2 CFE - Centre for Functional Ecology, Department of Life Sciences, University of Coimbra, Calçada Martim de Freitas, 3000-456 Coimbra, Portugal; 3 Secció de Bioquímica i Biologia Molecular, Departament de Bioquímica i Fisiologia, Facultat de Farmàcia i Ciències de l’Alimentació, Universitat de Barcelona (UB), Joan XXIII 27, 08028 Barcelona, Spain

## Abstract

In carnivorous fish, conversion of a glucose load to hepatic glycogen is widely used to assess their metabolic flexibility towards carbohydrate utilization, but the activities of direct and indirect pathways in this setting are unclear. We assessed the conversion of an intraperitoneal glucose load (2 g.kg^−1^) enriched with [U-^13^C_6_]glucose to hepatic glycogen in juvenile seabass and seabream. ^13^C-NMR analysis of glycogen was used to determine the contribution of the load to glycogen synthesis via direct and indirect pathways at 48-hr post-injection. For seabass, [U-^13^C_6_]glucose was accompanied by deuterated water and ^2^H-NMR analysis of glycogen ^2^H-enrichment, allowing endogenous substrate contributions to be assessed as well. For fasted seabass and seabream, 47 ± 5% and 64 ± 10% of glycogen was synthesized from the load, respectively. Direct and indirect pathways contributed equally (25 ± 3% direct, 21 ± 1% indirect for seabass; 35 ± 7% direct, 29 ± 4% indirect for seabream). In fasted seabass, integration of ^2^H- and ^13^C-NMR analysis indicated that endogenous glycerol and anaplerotic substrates contributed an additional 7 ± 2% and 7 ± 1%, respectively. In fed seabass, glucose load contributions were residual and endogenous contributions were negligible. Concluding, direct and indirect pathways contributed equally and substantially to fasting hepatic glycogen repletion from a glucose load in juvenile seabream and seabass.

## Introduction

European seabass (*Dicentrarchus labrax L*.) and gilthead seabream (*Sparus aurata L*.), are saltwater carnivorous fish that are widely farmed in the Mediterranean region. European production data for 2014 indicated a production of more than 145 000 tonnes for each species, which together represented around 13% of total European aquaculture fish production^1^. Given the increasing scarcity of fishmeal protein feed, there is current high interest in the development of alternative carbohydrate-based aquafeed ingredients that are less costly and more sustainable. Thus, there have been many studies into the capacities of seabass and seabream to adapt from their natural high-protein low-carbohydrate dietary regime to feeds with much higher carbohydrate to protein ratios^2–7^. The physiological response to a glucose load has been widely studied to assess the degree of metabolic flexibility of carnivorous fish species, including seabream and seabass, towards carbohydrate metabolism and utilization^8–11^. Conversion of the glucose load to hepatic glycogen is a key indicator of the metabolic capacity for assimilation of glucose carbons into the main pathways of hepatic intermediary metabolism. Glucose carbons can be converted to glycogen by two metabolic routes. The first is a linear pathway where the hexose skeleton remains intact and is commonly known as the direct pathway (the direct pathway refers to the conversion of glucose to glycogen via the following linear pathway: glucose → glucose-6-phosphate → glucose-1-phosphate → UDP-glucose → glycogen). The second route, known as the indirect pathway^12^, involves the initial conversion of glucose to pyruvate followed by gluconeogenic conversion of pyruvate to glucose-6-phosphate (G6P) and incorporation into glycogen via the same intermediates as for the direct pathway. The indirect pathway also provides a means of synthesizing hepatic glycogen from non-glucose precursors such as glycerol and gluconeogenic amino acids, which are abundant precursors in the typical high protein diets of carnivorous fish.

In seabass, analysis of hepatic glycogen position 5 enrichment from deuterated water (^2^H_2_O) was used to estimate indirect pathway contributions to glycogen synthesis^13^. With this approach, it was shown that essentially all hepatic glycogen synthesis during feeding with conventional fishmeal diet occurred via indirect pathway. However, this contribution could drop to ~70% if the fishmeal was partially replaced with digestible starch.

**Figure 1:**
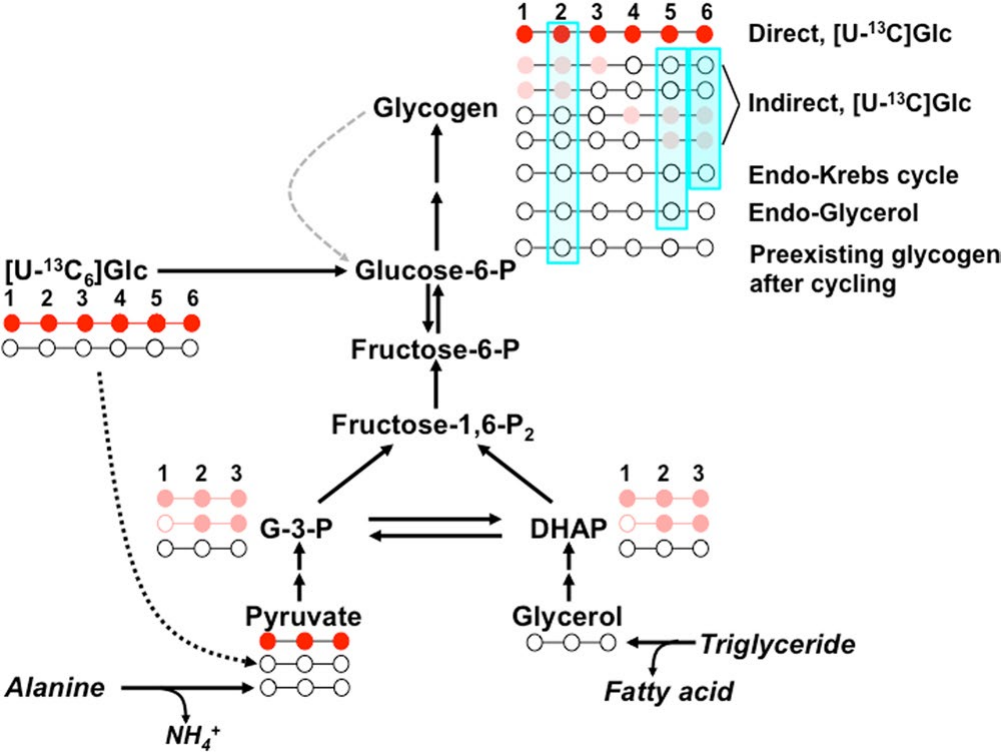
Schematic of liver glycogen ^2^H- and ^13^C-enrichment in seabass following administration of a glucose load enriched with [U-^13^C_6_]glucose ([U-^13^C_6_]Glc) while the fish are also in the presence of ^2^H-enriched tank water. The red circles represent glycogen ^13^C-isotopomers derived from direct pathway metabolism of [U-^13^C_6_]glucose and the pink circles indicate triose and glycogen isotopomers formed via indirect pathway metabolism of [U-^13^C_6_]glucose. The blue shading represents positional glycogen enrichment from ^2^H_2_O. This includes glycogen synthesized from alanine and other endogenous amino acids that are converted to glycogen via Krebs cycle anaplerosis and gluconeogenesis (Endo-Krebs cycle) as well as glycerol released by lipolysis (Endo-Glycerol). Pre-existing glycogen may also become enriched in position 2 from ^2^H_2_O via futile cycling of glycogen and glucose-6-phosphate (indicated by grey dashed arrow).

To our knowledge, direct and indirect pathway contributions to hepatic glycogen synthesis following a glucose load have not been reported for any fish species. This setting is expected to maximize direct pathway activity due to the high availability of glucose that in turn promotes insulin secretion^8^ and activation of hepatic glycogen synthesis^9^. A glucose load can be enriched with [U-^13^C_6_]glucose whose incorporation into hepatic glycogen can be used to independently measure direct and indirect pathway contributions of the glucose load to glycogen synthesis^14^. When [U-^13^C_6_]glucose is used in combination with ^2^H_2_O, indirect pathway contributions from non-glucose precursors can also be accounted for. NMR methodology can resolve glycogen ^2^H- and ^13^C-enrichment from ^2^H_2_O and [U-^13^C_6_]glucose allowing both tracers to be given simultaneously^14–16^. Taking this approach into consideration, we aimed at quantifying hepatic glycogen synthesis from a glucose load via both direct and indirect pathways in seabass and seabream by using an enriched glucose load with [U-^13^C_6_]glucose and quantifying the ^13^C-isotopomer distributions of glycogen. Also, we aimed at determining the contributions of both glucose and non-glucose precursors to hepatic glycogen synthesis in seabass following a glucose load by integrating [U-^13^C_6_]glucose and ^2^H_2_O tracers. The results generated from ^13^C-isotopomer and ^2^H-enrichment data were incorporated into a comprehensive metabolic model describing hepatic glycogen synthesis from all major substrate precursors, represented by Fig. 1.

## Materials and Methods

### Fish handling

Twenty four juvenile gilthead seabream (*S. aurata* L.) were maintained in two 200 L aquariums with recirculating water filtered by an external filter. Fish had a mean body weight of 27.6 ± 1.4 g. Fish were acclimated in a recirculating aquaculture system (RAS) equipped with a central filter and UV unit at 20 °C, 30‰ salinity and well aerated seawater (>90% O_2_). Water temperature, pH, dissolved oxygen and salinity were continuously monitored and nitrogenous waste products were measured twice weekly and were found to be within optimal ranges. During acclimation fish were fed daily until apparent satiety with a commercial diet (Dourasoja Ultra Energia 2, SORGAL, S.A.; 45.2% crude protein, 20.0% fat, 9.5% ash, 1.5% fiber, 1.0% phosphorus). Therefore, seabream were then fasted for 21 days.

Eighty juvenile European seabass (*D. labrax* L.), with body weight of 44.5 ± 3.1 g were randomly distributed into four 200 L tanks (n = 20 each). Fish were acclimated under the same conditions as previously described for seabream. Seabass were fed daily to apparent satiety with Aquagold 5 fish-feed, SORGAL, S.A. The diet was composed of 45.0% crude protein, 18.0% fat, 10.0% ash, 2.0% fiber, 2.0% calcium, 1.0% phosphorus, 0.4% sodium. After acclimation, two groups were fasted for 21 days while the others continued with the described feeding regime.

### Tracer delivery and sampling

At the end of the 21-day feeding/fasting period, both seabream and seabass were anesthetized in small seawater tanks containing 0.1 g.L^−1^ of MS-222. They were then measured and weighed and fish from half of the groups for each species were injected intraperitoneally with 2 g.kg^−1^ glucose enriched to 10.0–12.5% with [U-^13^C_6_]glucose and transferred back to the main rearing tanks for 48 hours without feeding. For the seabass, the seawater was also enriched to 3.5% with ^2^H_2_O during the 48 hour post-injection period. Fish from the other half of the groups were injected with saline to assess hepatic glycogen levels in the absence of a glucose load. After this period, the fish were again anesthetized in seawater tanks containing 0.1 g.L^−1^ of MS-222 and also enriched to 3.5% with ^2^H_2_O in the case of seabass. Fish were measured, weighed and livers were extracted and freeze clamped in liquid nitrogen and then stored at −80 °C until further processing. After sacrifice by cervical section perivisceral fat was collected and weighed. Enriched seawater with ^2^H_2_O from the seabass was collected to confirm its enrichment. All the above procedures were in compliance with the Guidelines of the European Union Council (2010/63/EU) after approval by the Animal Care Committee of the University of Coimbra.

### Hepatic glycogen extraction and derivatization

In order to insure glycogen samples that yielded ^2^H- and ^13^C-NMR spectra with high signal-to-noise ratios, livers from 2-3 fish were pooled. Glycogen extraction, quantification, hydrolysis to glucose and derivatization to monoacetone glucose (MAG) was as previously described^13^. MAG was further purified by solid phase extraction using Discovery^®^ DSC-18 3 mL/500 mg disposable columns (Sigma-Aldrich). Columns were activated with 3 mL acetonitrile followed by 10 mL distilled water. The MAG preparation was dissolved in 1 mL distilled water and added to the column. The column was then washed with 0.5 mL distilled water to remove impurities and the MAG was eluted with 2.5 mL of 10/90 v/v acetonitrile/water and the solvent then evaporated.

### NMR spectroscopy and analysis

Proton-decoupled ^13^C-NMR spectra of MAG derived from seabream were acquired at 14.1 T with a Varian VNMRS 600 MHz NMR (Agilent, Santa Clara, CA, USA) spectrometer equipped with a 3 mm broadband probe with *z*-gradient. Spectra were obtained at a temperature of 25 °C, using a 70° pulse, 2.5 s acquisition time, 0.5 s pulse delay and 5,000 to 15,000 free-induction decays (f.i.d.). ^13^C-NMR spectra of MAG derived from seabass were obtained with a Bruker Avance III HD 500 (Bruker Biospin, Billerica, MA, USA) spectrometer equipped with a BBFO 5 mm broadband probe using the same pulse parameters and f.i.d. numbers. Seabass ^13^C NMR spectra were obtained following ^2^H-NMR acquisition and evaporation of the acetonitrile/water solvent mixture (see below) and resuspension of the MAG residue in 0.5 ml 99.9% ^2^H_2_O. Quantification of the glycogen ^13^C-isotopomers representing direct pathway and indirect pathway conversion of [U-^13^C_6_]glucose to glycogen was performed by deconvolution of the MAG carbon 1 resonance multiplet as previously described^14,17^. The two methyl natural-abundance carbon signals of MAG were used as intramolecular standards^18^ for converting the carbon 1 ^13^C isotopomer multiplet areas into ^13^C-enrichment values.

Proton-decoupled ^2^H-NMR spectra of MAG samples obtained from seabass were also obtained with a Bruker Avance III HD 500 spectrometer using a ^2^H-selective 5 mm probe incorporating a ^19^F-lock channel. Samples were resuspended in 0.5 ml 90% acetonitrile/10% ^2^H-depleted water to which 50 μl of hexafluorobenzene were added. ^2^H-NMR spectra were obtained with a 90° pulse angle, 1.6 s of acquisition time and a 0.1 s interpulse delay. The number of f.i.d. collected ranged from 5,000-20,000. Positional ^2^H-enrichments were determined using the MAG methyl signals as an intramolecular standard^19^. To quantify fish plasma body water and seawater ^2^H-enrichments, triplicate 10 µL samples of plasma were analyzed by ^2^H NMR as previously described^20^ but with 50 μl of hexafluorobenzene added to the NMR sample. ^13^C- and ^2^H-NMR spectra were analyzed with ACD/NMR Processor Academic Edition software (ACD/Labs, Advanced Chemistry Development, Inc.).

### Theory and metabolic flux calculations

#### ^13^C Glycogen enrichment from [U-^13^C_6_]glucose

Analysis of glycogen ^13^C-isotopomer distributions after an intraperitoneal injection of [U-^13^C_6_]glucose informs the fractional rates of glycogen production from the glucose load via both direct and indirect pathways^14,21^. Direct pathway conversion of glucose to glycogen via G6P does not cleave the hexose carbon skeleton thus [U-^13^C_6_]glucose is converted into [U-^13^C_6_]glucosyl units of glycogen. The direct pathway fractional contribution is calculated according to the following equation:

1 $${\rm{Direct}}\,{\rm{pathway}}\,( \% )=[{{\rm{U}} \mbox{-} }^{{\rm{13}}}{{\rm{C}}}_{{\rm{6}}}]{\rm{glycogen}}\,{\rm{enrichment}}\times {\rm{100}}/{{\rm{F}}}_{{\rm{g}}}$$

F_g_ represents the percent [U-^13^C_6_]glucose enrichment of the glucose load.

The indirect pathway metabolism of glucose to glycogen involves its initial conversion to pyruvate via glycolysis followed by the re-formation of G6P via anaplerosis and gluconeogenesis and subsequent conversion to glycogen. During this sequence, the [U-^13^C_6_]glucose skeleton is cleaved and the ^13^C-label also undergoes both dilution and randomization to form partially-labeled glycogen ^13^C-isotopomers distributed within each triose half (see Fig. 1). The most prevalent are [1,2-^13^C_2_]- and [1,2,3-^13^C_3_]glycogen, with corresponding [5,6-^13^C_2_]- and [4,5,6-^13^C_3_]glucosyl isotopomers from the other triose half ^22^. Assuming that the two triose halves have equivalent ^13^C-isotopomer distributions, the fractional contribution of the indirect pathway to the glycogen pool can be calculated from the C123 triose moiety – whose component ^13^C-isotopomers have better-resolved ^13^C-NMR signals – as follows.

2 $$\begin{array}{rcl}{\rm{Indirect}}\,{\rm{pathway}}\,{\rm{from}}\,{\rm{glucose}}\,{\rm{load}}\,( \% ) & = & [{\rm{1}},{2 \mbox{-} }^{{\rm{13}}}{{\rm{C}}}_{{\rm{2}}}]{\rm{glycogen}}\\  &  & +[{\rm{1}},{\rm{2}},{3 \mbox{-} }^{{\rm{13}}}{{\rm{C}}}_{{\rm{3}}}]{\rm{glycogen}}\times {\rm{1.5}}\times {\rm{100}}/{{\rm{F}}}_{{\rm{g}}}\end{array}$$

The factor of 1.5 accounts for dilution of ^13^C enrichment at the level of the Krebs cycle and is based on the assumption that net anaplerotic outflow is ~2-fold that of Krebs cycle oxidative flux, as has been measured in both rodents^23^ and humans^24^.

### ^2^H-enrichment of glycogen from ^2^H_2_O

Glycogen hexose units that has been synthesized in the presence of ^2^H_2_O becomes enriched in specific positions as a result of water hydrogen exchange and/or addition to metabolic intermediates catalyzed by specific enzymes of the glycogen synthesis pathways^13,25–27^ as shown in Fig. 1. The ^2^H-enrichment of glycogen position 5 (H5) relative to tank water provides an estimate of the fractional contribution of all indirect pathway sources to the glycogen pool.

3 $${\rm{All}}\,{\rm{indirect}}\,{\rm{pathway}}\,{\rm{sources}}\,( \% )=100\times {\rm{H}}5/{\rm{tank}}\,{\rm{water}}$$

Glycogen synthesized from Krebs cycle precursors, including indirect pathway metabolism of the glucose load, is enriched in both positions 5 and 6 _*S*_ while glycogen derived from substrates that enter glycogenesis at the level of triose phosphates, such as glycerol, is only enriched in position 5. Thus, indirect pathway contributions from the Krebs cycle are calculated from the 6 _*S*_ enrichment relative to tank water as follows:

4 $${\rm{Contribution}}\,{\rm{from}}\,{\rm{all}}\,{\rm{Krebs}}\,{\rm{cycle}}\,{\rm{substrates}}\,( \% )=100\times {\rm{H}}{6}_{S}/{\rm{tank}}\,{\rm{water}}$$

Contributions from glycerol released via lipolysis are estimated as the difference between position 5 and 6_*S*_ enrichments relative to tank water:

5 $${\rm{Contribution}}\,{\rm{from}}\,{\rm{endogenous}}\,{\rm{glycerol}}\,( \% )=100\times ({\rm{H}}5-{\rm{H}}{6}_{{S}})/{\rm{tank}}\,{\rm{water}}$$

### Integration of ^2^H_2_O and [U-^13^C]glucose tracer analyses

The fraction of glycogen that was derived via indirect pathway metabolism of endogenous Krebs cycle precursors other than the glucose load (for example alanine or other gluconeogenic amino acids) was calculated as the difference between total Krebs cycle contribution (Equation 4) and the specific contribution from the glucose load (Equation 2).

6 $$\begin{array}{rcl}{\rm{Indirect}}\,{\rm{endogenous}}\,{\rm{Krebs}}\,{\rm{cycle}}\,( \% ) & = & {\rm{Total}}\,{\rm{Krebs}}\,{\rm{cycle}}\,{\rm{contribution}}\\  &  & -\mathrm{Indirect}\,{\rm{pathway}}\,{\rm{glucose}}\,{\rm{load}}\,{\rm{contribution}}\end{array}$$

The fraction of hepatic glycogen that was synthesized during the period that the fish were administered with [U-^13^C_6_]glucose and ^2^H_2_O tracers, defined as newly synthesized glycogen, was calculated as follows:

7 $$\begin{array}{rcl}{\rm{Newly}}\,{\rm{synthesized}}\,{\rm{glycogen}}\,( \% ) & = & {\rm{Direct}}\,{\rm{pathway}}\,{\rm{fraction}}\\  &  & +\,{\rm{all}}\,{\rm{indirect}}\,{\rm{pathway}}\,{\rm{fractions}}\end{array}$$

Finally, the fraction of pre-existing hepatic glycogen was estimated as the balance between total and newly synthesized glycogen fractions.

8 $$\mathrm{Pre} \mbox{-} \mathrm{existing}\,{\rm{fraction}}\,( \% )=100\,-\,{\rm{Newly}}\,{\rm{synthesized}}\,{\rm{fraction}}$$

Fractional pathway contributions were converted to absolute values by multiplication with liver glycogen amounts, expressed as grams per 100 grams liver wet weight.

### Statistical analysis

Values are expressed as means ± standard error of the mean (S.E.M.). Statistical differences between different groups with *P* < 0.05 were assessed using Student’s two-tailed unpaired *t* test.

### Data availability

The datasets generated and analysed during the current are available from the corresponding author on reasonable request.

## Results

### Hepatic metabolite levels

There were no differences in weights between seabass groups (see Table 1). The hepatosomatic index (HSI) was significantly different between fed and fasted seabass as well as between fasted seabass and fasted seabream (*P* < 0.0001 for both). Fed and fasted seabass also had significantly different for perivisceral fat somatic index (PFSI) (*P* < 0.05). This index could not be calculated for seabream since they had no observable perivisceral fat. Similar differences were also observed for hepatic glycogen levels with significantly lower amounts in fasted compared to fed seabass (*P* < 0.05). Hepatic glycogen levels were also significantly different between fasted seabream and fasted seabass (*P* < 0.0005, respectively). Compared to our previous study with 90 g fasted seabass^9^, liver glycogen levels were somewhat higher: 8.4 ± 0.5 g.100 g^−1^ liver in this study *versus* 6.6 ± 0.6 g.100 g^−1^ liver previously. From a comparison of hepatic glycogen levels in saline-injected *versus* glucose-injected seabass, the glucose load resulted in a significant increase in hepatic glycogen levels of fasted fish while it had no significant effects on glycogen levels of fed fish (Table 1).

**Table 1:** Physiological parameters and hepatic glycogen levels for fed and fasted seabass and fasted seabream 48 hours after the administration of a glucose load. Also shown are hepatic glycogen levels for seabass that received a saline injection instead of a glucose load. Each n for liver glycogen represents a pooled sample from livers of 2–3 fish.

	Seabass		Seabream
	Fed	Fasted	Fasted
Body weight, (g)	44.5 ± 3.1 (n = 40)	38.9 ± 2.4 (n = 40)	27.6 ± 1.4 (n = 24)
^1^HSI	2.5 ± 0.2^**^ (n = 19)	1.4 ± 0.1 (n = 14)	0.71 ± 0.03 ^**^ (n = 12)
^2^PFSI	8.1 ± 0.7^*^ (n = 10)	5.6 ± 0.6 (n = 10)	Not determined^3^
Final liver glycogen (g.100 g^−1^ liver)	10.0 ± 0.5^*^ (n = 6)	8.4 ± 0.5 (n = 6)	1.6 ± 0.7 ^**^ (n = 3)
Saline load final liver glycogen (g.100 g^−1^ liver)	10.4 ± 0.2^**^ (n = 6)	3.4 ± 1.1^**‡‡**^ (n = 6)	0.2 ± 0.01 (n = 3)

Values are means ± S.E.M. Significant differences between different seabass feeding conditions and between different species in fasted state (except weight) are indicated by asterisks (*t*-test, *P < 0.05; **P < 0.001). Significant differences between Saline load and Final liver glycogen are indicated with ^**‡**^(*t*-test, ^**‡‡**^*P* < 0.005). ^1^Hepatosomatic index = 100 × (liver weight/body weight).

^2^Perivisceral fat somatic index = 100 × (perivisceral fat weight/body weight).

^3^Absence of perivisceral fat.

### Hepatic glycogen ^13^C-enrichment from the [U-^13^C_6_]glucose load and estimates of direct and indirect pathway contributions to glycogen synthesis in fasted seabream and seabass

**Figure 2:**
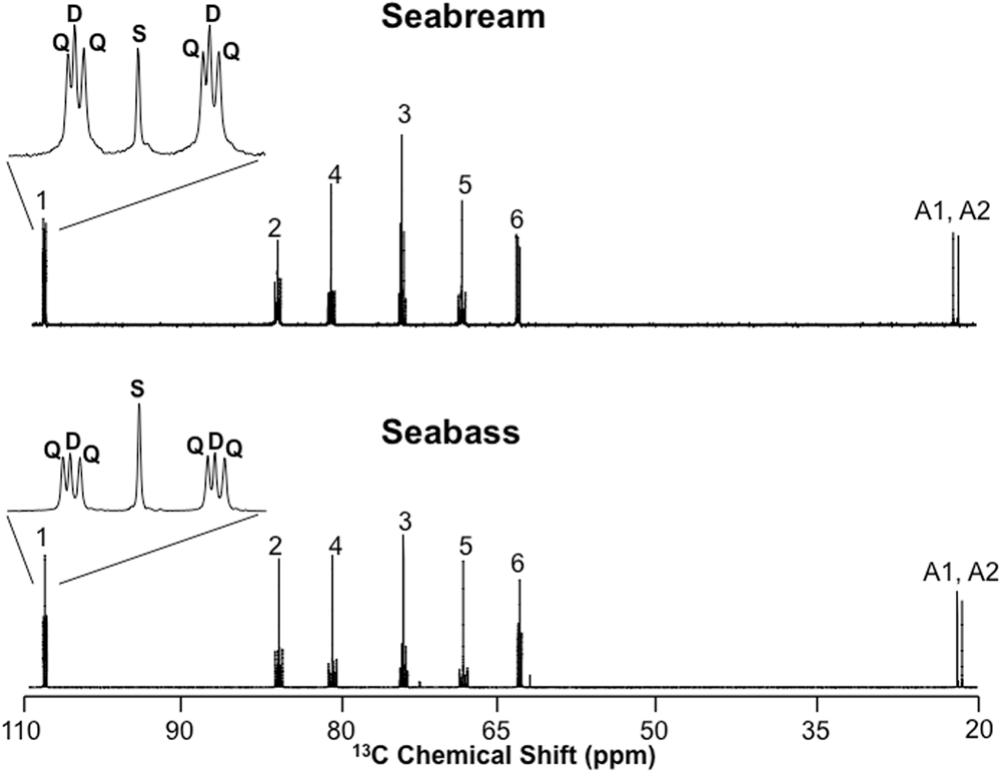
^13^C NMR spectrum of the liver glycogen MAG derivative from fasted seabream (top) and fasted seabass (bottom) following administration of a glucose load enriched with [U-^13^C]glucose. The numbers above each resonance indicates its position in the glucosyl hexose unit. The signals marked A1, A2 are the two methyl carbons of the MAG derivative. For both spectra, the carbon 1 resonance is shown in expanded form to illustrate the **Q, D** and **S** multiplet components arising from direct and indirect pathway metabolism of [U-^13^C]glucose to glycogen, and background ^13^C, respectively.

**Figure 3:**
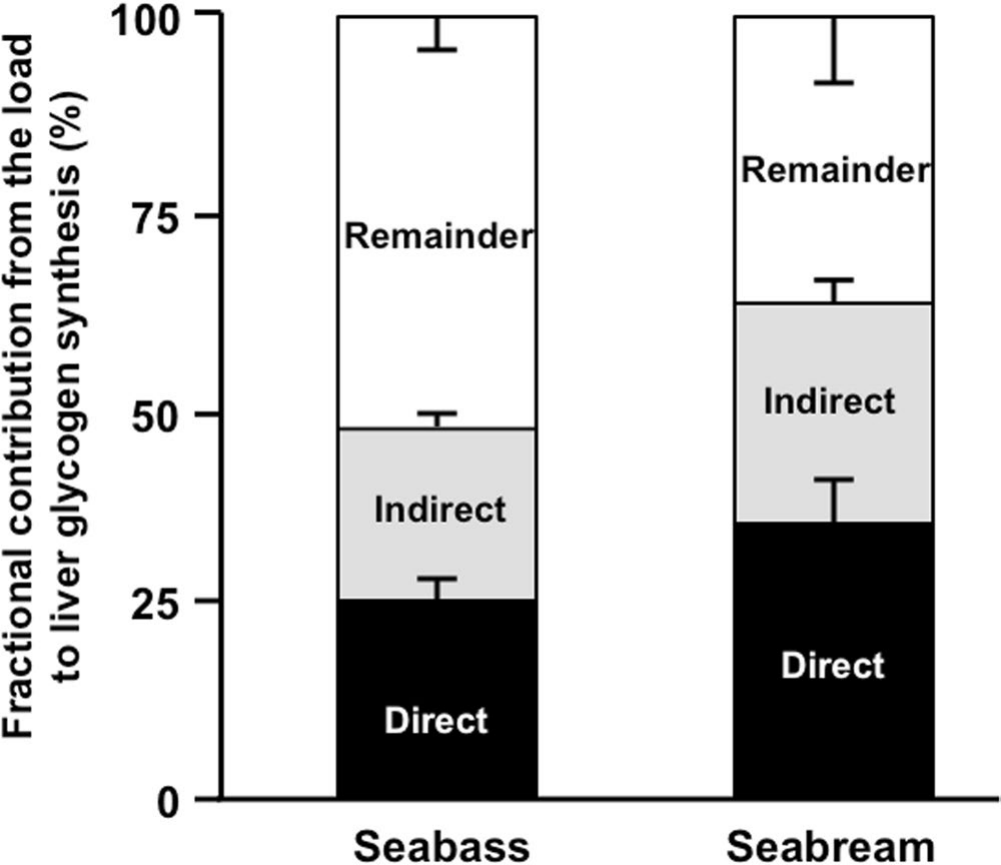
Fraction of hepatic glycogen derived from an intraperitoneal glucose load at 48 hours post-load in fasted seabream and seabass. The contributions of direct and indirect pathways of glycogen synthesis to the synthesized fraction are also shown. The remainder represents pre-existing glycogen and/or glycogen synthesized from unlabeled precursors.

Enrichment of the load with [U-^13^C_6_]glucose generates glycogen ^13^C-isotopomers that can be attributed to direct and indirect pathway activities, as shown in Fig. 1. Figure 2 illustrates the identification of direct and indirect pathway ^13^C-isotopomers by ^13^C-NMR spectroscopy of glycogen following its derivatization to MAG. Each of the six hexose signals is composed of a central singlet arising from the background ^13^C and flanking multiplet signals that originate from the [U-^13^C]glucose precursor. The pair of upfield singlet signals (A1, A2) are from the two methyl carbons of MAG that are incorporated from acetone during the derivatization process and therefore do not have any ^13^C-enrichment from metabolic activity. Hence, they serve as convenient intramolecular ^13^C-enrichment standards for quantifying ^13^C-enrichment of the hexose-derived signals^18^. The carbon 1 resonance, shown in expanded form, consists of a central singlet signal from background ^13^C that is flanked by a well resolved quartet (**Q)** component from [U-^13^C_6_]glucosyl and a doublet (**D)** representing the sum of [1,2-^13^C_2_]- and [1,2,3-^13^C_3_]glucosyl units. As shown in Fig. 1, the [U-^13^C_6_]glucosyl isotopomer represents direct pathway metabolism of [U-^13^C_6_]glucose to glycogen while the [1,2-^13^C_2_]- and [1,2,3-^13^C_3_]glucosyl isotopomers correspond to [U-^13^C_6_]glucose incorporation into glycogen via the indirect pathway. Inspection of the carbon 5 and 6 multiplets indicated that the [1,2-^13^C_2_]- and [1,2,3-^13^C_3_]glucosyl isotopomers were accompanied by equivalent amounts of [5,6-^13^C_2_]- and [4,5,6-^13^C_3_]glucosyls - as would be expected from extensive exchange of the triose-P precursors catalyzed by triose phosphate isomerase (data not shown). The prominence of the **D** signals in both seabream and seabass spectra indicate that the indirect pathway had a significant role in the conversion of the glucose load to hepatic glycogen for both species. Table 2 shows estimates of ^13^C-isotopomer enrichments obtained from the ^13^C-NMR spectra corresponding to direct and indirect pathway activities. From these data, the fraction of hepatic glycogen derived from direct and indirect pathway metabolism of the glucose load were estimated using Equations (1) and (2) from the materials and methods section and the results are represented by Fig. 3. The data demonstrate that a substantial portion of hepatic glycogen (~50% for seabass and ~65% for seabream) was derived from the glucose load with near-equivalent contributions from direct and indirect pathways. The remaining unlabeled fraction represents glycogen that was synthesized from unlabeled precursors and/or pre-existing glycogen.

**Table 2:** Liver glycogen ^13^C-enrichment, selected ^13^C-isotopomer abundances, selected positional ^2^H-enrichments, tank water ^2^H-enrichment and the ratio of glycogen position 2 to tank water enrichment (H2/TW) for groups of fed and fasted seabass 48 hours following administration of a glucose load enriched with [U-^13^C]glucose in the presence of tank water enriched with ^2^H_2_O. Also shown are glycogen ^13^C-enrichment and selected ^13^C-isotopomer distributions for a group of fasted seabream administered with [U-^13^C]glucose only. Each value represents a mean with its S.E.M. shown below in parentheses. Each sample represents glycogen pooled from the livers of 2–3 fish.

	Glycogen ^13^C-enrichment and isotopomer distributions from [U-^13^C]glucose (%)			Glycogen positional ^2^H-enrichment from ^2^H_2_O (%)			Tank water ^2^H-enrichment	H2/TW (%)
	C1 excess enrichment	[U-^13^C_6_]Glycogen	[1,2,3-^13^C_3_]- + [1,2-^13^C_2_]Glycogen	Position 2	Position 5	Position 6 _*S*_		
Fed Seabass (n = 9 samples)	0.39^*^ (0.09)	0.19^*^ (0.05)	0.20^*^ (0.05)	0.27^a*^ (0.02)	0.11^*^ (0.01)	0.08^*^ (0.01)	3.66 (0.27)	6.8 (1.1)
Fasted Seabass (n = 7 samples)	4.94 (0.52)	3.16 (0.43)	1.78 (0.09)	3.04^a^ (0.07)	1.20 (0.04)	0.96 (0.04)	3.43 (0.03)	88.5 (3.0)
Fasted Seabream (n = 4 samples)	6.78 (1.21)	4.39 (0.92)	2.38 (0.33)	N.D.	N.D.	N.D.	N.D.	N.D.

Values are means (S.E.M.).Significant differences between different feeding conditions are indicated by asterisks (*t*-test, *P < 0.0001). No significant differences between different species in fasted state were found.

^a^Values are corrected for incomplete exchange of position 2 and body water (Martins *et al*., 2013a).

### Hepatic glycogen synthesis from all sources in seabass using [U-^13^C_6_]glucose and ^2^H_2_O

**Figure 4:**
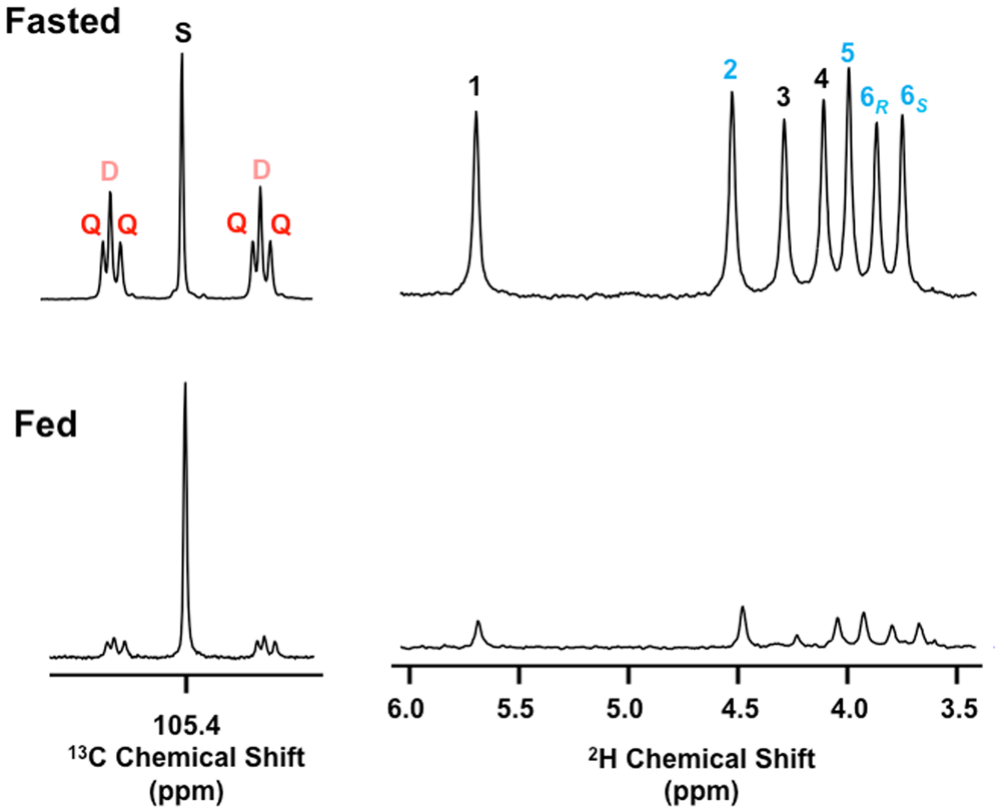
Selected ^13^C NMR and ^2^H NMR signals of the liver glycogen MAG derivative from 21-day fasted and from fed seabass following administration of a glucose load enriched with [U-^13^C]glucose in the presence of ^2^H-enriched tank water. On the left-hand side are shown the carbon 1 multiplet from the ^13^C NMR spectra for each condition with **S**, **D** and **Q** notations as described in Fig. 2. On the right-hand side are shown the corresponding ^2^H NMR spectra with the number above each signal representing its position in the glycogen hexose unit. The ^13^C and ^2^H NMR glycogen signals of fasted and fed fish were scaled by matching the intensities of the ^13^C and ^2^H MAG methyl signals (not shown) which represent constant ^2^H and ^13^C enrichment levels across the entire set of samples.

When [U-^13^C_6_]glucose is combined with ^2^H_2_O, glycogen synthesis from the glucose load and from non-glucose precursors can be resolved. Also, the fraction of pre-existing glycogen can be estimated. This is particularly relevant in carnivorous fish under fed conditions where endogenous glycogenic precursors such as gluconeogenic amino acids may be abundant, and pre-existing hepatic glycogen stores are also plentiful. Figure 4 shows ^13^C- and ^2^H-NMR spectra taken from liver samples of fasted and fed seabass. In the ^2^H NMR spectrum, the position 2 intensity appears to be less than that of position 5 - consistent with a previous seabass study of glycogen enrichment from ^2^H_2_O^13^. This is due to a significant kinetic isotope effect that limits the exchange between the hydrogen 2 of G6P and body water^9^ thereby decreasing the enrichment of this specific site from ^2^H_2_O. The magnitude of this effect can be quantified from the analysis of glycogen ^2^H-enrichment from [U-^2^H_7_]glucose allowing the position 2 enrichment from ^2^H_2_O to be corrected for incomplete exchange^9^ (see Table 2).

The ^13^C-multiplet intensities of the ^13^C-NMR spectra of the fed fish were substantially lower compared to fasted fish indicating a much smaller enrichment of glycogen from [U-^13^C_6_]glucose in fed compared to fasted fish (see Table 2). The ^2^H NMR signals and estimated ^2^H-enrichment levels, which inform glycogen synthesis from both [U-^13^C_6_]glucose and endogenous substrates, were also much less in fed *versus* fasted fish. Integration of ^2^H- and ^13^C-NMR data informs the fraction of glycogen synthesized from all sources via direct and indirect pathways, and these data are presented in Table 3.

**Table 3:** Percentage contributions of direct and indirect pathway substrates to hepatic glycogen synthesis in seabass under fed (n = 9) and fasted (n = 7) states following the administration of a glucose load enriched with [U-^13^C]glucose and in the presence of tank water enriched with ^2^H_2_O. Each n represents glycogen pooled from livers of 2–3 fish. Indirect pathway contributions are resolved into glucose load, endogenous Krebs cycle substrates (Endo-Krebs) and endogenous glycerol (Endo-Glycerol). For each metabolic flux parameter, the corresponding equation is also shown.

	Direct pathway (Eq. 1)	Indirect pathway (Eq. 3)			Newly Synthesized (Eq. 7)	Pre-existing (Eq. 8)
		Glucose load (Eq. 2)	Endo-Krebs (Eq. 6)	Endo-Glycerol (Eq. 5)		
**Fed seabass**	1.5 ± 0.4^**^	2.4 ± 0.6^**^	−0.5 ± 0.4 ^*^	0.9 ± 0.2^**^	4.0 ± 1.0^**^	96.0 ± 0.8^**^
**Fasted seabass**	25.3 ± 3.4	21.3 ± 1.1	6.5 ± 2.3	7.1 ± 0.7	60.2 ± 3.1	39.8 ± 3.1

Values are means ± S.E.M. Significant differences between different feeding conditions are indicated by asterisks (*t*-test, *P < 0.05; **P < 0.001).

These results show that under fed conditions, the fraction of hepatic glycogen derived from the glucose load was very small. Of this small fraction, the indirect pathway had a substantial contribution, being roughly equivalent to that of the direct pathway. The majority of indirect pathway carbons and essentially all Krebs cycle sources were accounted for by the glucose load. Under fasted conditions, the fraction of newly synthesized glycogen was far more substantial, accounting for 60% of the total glycogen sampled at 48 hours post load. While about 2/3^rds^ of the newly synthesized glycogen was derived from the glucose load - with approximately equal contributions from direct and indirect pathways - there were also meaningful contributions from endogenous Krebs cycle and glycerol sources. Absolute values of glycogen synthesis from the glucose load and endogenous sources, obtained by multiplying fractional rates by the total glycogen concentration, are shown in Table 4. These data reveal a ~10–15-fold higher uptake of glucose into glycogen under fasted compared to fed conditions. Glycogen synthesis from endogenous sources was also significantly higher in the fasted state.

**Table 4:** Contributions of direct and indirect pathway substrates to liver glycogen synthesis in absolute values (g.100 g^−1^ liver) in fed (n = 9) and fasted (n = 7) seabass following the administration of a glucose load enriched with [U-^13^C]glucose and in the presence of tank water enriched with ^2^H_2_O. Each n represents glycogen pooled from livers of 2–3 fish.

	Direct pathway	Indirect pathway			Newly Synthesized	Pre-existing
		Glucose load	Endo-Krebs	Endo-Glycerol		
Feeding seabass	0.15 ± 0.04^**^	0.24 ± 0.05^**^	−0.05 ± 0.04^*^	0.09 ± 0.01^**^	0.40 ± 0.10^**^	9.52 ± 0.08^**^
Fasting seabass	2.12 ± 0.29	1.79 ± 0.09	0.55 ± 0.19	0.59 ± 0.06	5.04 ± 0.26	3.33 ± 0.26

Values are means ± S.E.M. Significant differences between different feeding conditions are indicated by asterisks (*t*-test, *P < 0.05; **P < 0.001).

These results are also in good agreement with our biochemical estimates of hepatic glycogen excursions based on hepatic glycogen quantification of fish administered with glucose and saline loads (Table 1). For the fed seabass, the glucose load did not significantly alter the levels of hepatic glycogen compared to saline. For the fasted seabass, the difference in glycogen levels between saline and glucose loads (~5.6 g.100 g^−1^ liver) corresponds to ~67% of the total glycogen sampled. This is consistent with the 60% fraction of newly synthesized glycogen calculated from the tracer studies.

## Discussion

### Rationale of our study design and setting

The conversion of glucose to glycogen by the liver is a key process to define the capacity of an organism to utilize dietary glucose. Thus, the rate of hepatic glycogen synthesis is widely used as a proxy for glucose utilization in many different organisms including fish^3,4,9,10,13,28^. Resolution of hepatic glycogen synthesis into direct and indirect pathway contributions gives further insight on the roles and capacities of these pathways in mediating the conversion of glucose to glycogen. Many studies have shown that hepatic glycogen repletion in fish is most active when they are presented with food or with a glucose bolus after fasting^13,29–31^. Quantifying direct and indirect pathway activities in this setting, where glucose availability is not a limiting factor, provides valuable insight on the capacity of each pathway in sustaining hepatic glycogen synthesis.

### Comparison of direct and indirect pathway activities of seabass and seabream with other species

In omnivorous species such as humans and rats that are considered to be well adapted to assimilate and metabolize glucose, the indirect pathway contributes significantly to hepatic glycogen re-synthesis after fasting induced by a carbohydrate-containing meal or a glucose load. In rats, the indirect pathway accounts for 50–70% of glycogen incorporation from a glucose load^14,21^ and ~54% of hepatic glycogen repletion during nocturnal feeding with standard chow^26^. In a model of insulin-dependent (Type 1) diabetes induced by streptozotocin, the indirect pathway contribution was significantly higher (68%) but could be restored to the levels found in non-diabetic controls by insulin administration^32^. In healthy humans, the indirect pathway accounts for 30–50% of hepatic glycogen synthesis following a meal or a glucose load^33–35^. As with rats, this contribution is significantly increased in Type 1 diabetes^34,36^ but it does not revert to normal following insulin therapy^33,36^. Given that carnivorous fish have been previously compared to glucose-intolerant or diabetic mammalian species in their poor capacity to clear a glucose load^37^, and based on our previous measurements of high indirect pathway activities in seabass fed with either standard or starch-supplemented aquafeeds^7,13^ we anticipated that the indirect pathway would dominate hepatic glycogen synthesis from a glucose load for seabass and seabream. Instead, we found that the direct pathway contributed between one-third and one-half of hepatic glycogen synthesis from glucose – similar to that of a healthy rat. These results suggest that the capacity for hepatic glucose metabolism in seabass and seabream is comparable to that of omnivorous mammals and that their glucose intolerance may be more related to peripheral glucose uptake and metabolism or/and inability to shut down gluconeogenesis even if glycolysis and glycogenesis seem well regulated after glucose load. Also, the fact that a glucose load appears to sustain higher direct pathway contributions compared to that measured after feeding with aquafeed enriched in dietary starch^7^ suggests that polysaccharide digestion may be a limiting step in the supply of glucose for direct pathway flux in carnivorous fish.

### Role of extrahepatic glucose metabolism in sustaining indirect pathway flux

The indirect pathway of glucose conversion to hepatic glycogen involves the initial glycolytic conversion of glucose to pyruvate followed by the gluconeogenic conversion of pyruvate to G6P. In the whole body, glucose can be converted to pyruvate in peripheral tissues such as muscle and this pyruvate can be then exported to the liver (usually in the form of lactate or alanine) to be converted into G6P. Conversion of this G6P to glucose completes the Cori and/or glucose-alanine cycle. If this G6P is recruited for glycogen synthesis, it is tantamount to the conversion of glucose to glycogen via the indirect pathway. Tracer methods cannot distinguish this form of indirect pathway flux involving extrahepatic glucose metabolism from that conducted solely within the liver. Therefore, it is not known to what extent peripheral tissues contribute to the indirect pathway formation of hepatic glycogen from a glucose load. We do know that there is extensive Cori cycling of a glucose load in seabass^11^ hence it is likely that a significant portion of indirect pathway flux involves extrahepatic glucose metabolism.

### Glycogen cycling and its impact on ^2^H_2_O measurements of direct pathway flux

Futile cycling between glycogen and G6P has been described in humans under certain nutritional conditions or disease states^38–40^ and was also found to be active in rats following a glucose load^41^. With ^2^H_2_O, futile cycling contributes to the enrichment of glycogen position 2 (H2) independently of net glycogen synthesis thereby confounding direct pathway estimates^27,34,39^. Glycogen cycling does not influence the enrichment of position 5 (H5) hence estimates of indirect pathway contributions based on the H5/body water enrichment ratio are unaffected. For determining the extent of glycogen cycling, the ratio of glycogen position 2 to tank water enrichment (H2/TW), representing the fraction of newly synthesized glycogen plus the fraction of glycogen that underwent cycling, is compared with the estimate of newly synthesized glycogen obtained by mass balance^39^. Based on our biochemical measurements of hepatic glycogen in glucose- and sham-injected fish, we estimated that 67% of the post-load glycogen was newly-synthesized. From H2/TW (see Table 2), the fraction of newly-synthesized plus cycled glycogen was 88%. Thus the difference, 21%, can be attributed to glycogen cycling. This additional labeling of position 2 represents the conversion of unlabeled pre-existing glycogen to [2-^2^H]glycogen via glycogen cycling (see Fig. 1). Since pre-existing glycogen constituted 33% of the total, the fraction of glycogen that underwent cycling can be estimated as 21/33 = 64%. Thus in seabass administered with a glucose load, hepatic glycogen cycling appears to be very extensive. As a result, analysis of glycogen would have yielded substantial overestimates of direct pathway contributions to hepatic glycogen synthesis (53% *versus* 25% calculated using [U-^13^C_6_]glucose).

### Effects of pentose phosphate pathway activity on flux estimates

The oxidative portion of the pentose phosphate pathway (PPP) results in the loss of carbon 1 while the non-oxidative portion results in carbon rearrangements through transaldolase and transketolase activities. Therefore, both oxidative and non-oxidative PPP activity will alter glycogen enrichment distributions from ^2^H- and ^13^C-tracers. Such activity is revealed most clearly in the ^13^C-isotopomer data^42,43^. In the absence of PPP flux, the ^13^C-isotopomers derived from indirect pathway metabolism have identical distributions in the 123- and 456- carbons of glycogen. If PPP flux is active, the 123- carbons undergo significant rearrangement while the 456- moiety remains intact^42^. Analysis of the carbon 2 and carbon 5 ^13^C signals from our experiments (data not shown) showed near-identical 123- and 456- isotopomer distributions indicating that PPP flux was low in relation to G6P flux into glycogen. These observations are consistent with our previous studies of seabass glycogen enrichment from a glucose load enriched with [U-^2^H_7_]glucose where the effects of transaldolase exchange activity on glycogen ^2^H-enrichment distributions were found to be insignificant^9^.

### Conclusions

There is high interest in understanding the degree to which carnivorous fish such as seabass and seabream can adapt to dietary carbohydrate. Since the hepatic conversion of glucose to glycogen is considered a central pathway in carbohydrate assimilation, we studied this process using stable-isotope tracers that allowed glycogen synthesis from both an exogenous glucose load as well as endogenous non-glucose substrates to be measured. Moreover, the overall conversion of glucose into hepatic glycogen was resolved into direct and indirect pathway contributions. We found that for both fasted seabream and seabass, hepatic glycogen was efficiently replenished by a glucose load. This repletion involved approximately equal contributions of direct and indirect pathways, which is similar to that observed in rodents and in humans. In our study of hepatic glycogen repletion from glucose in fasted seabass using a combination of [U-^13^C_6_]glucose and ^2^H_2_O, we found evidence of substantial substrate cycling between glycogen and G6P. Since this activity can result in the enrichment of glycogen position 2 from ^2^H_2_O without the net synthesis of new glycogen, analysis of direct pathway contributions to hepatic glycogen synthesis based on position 2 enrichment can result in significant overestimates. These uncertainties may be avoided by using a combination of ^2^H_2_O and [U-^13^C_6_]glucose tracers.
