# Author Correction: Disposition of a Glucose Load into Hepatic Glycogen by Direct and Indirect Pathways in Juvenile Seabass and Seabream

**DOI:** 10.1038/s41598-018-24104-9

**Published:** 2018-04-13

**Authors:** João Rito, Ivan Viegas, Miguel A. Pardal, Isidoro Metón, Isabel V. Baanante, John G. Jones

**Affiliations:** 1 CNC - Center for Neuroscience and Cell Biology, Rua Larga, 1° Piso da FMUC, University of Coimbra, 3004-504 Coimbra, Portugal; 2 CFE - Centre for Functional Ecology, Department of Life Sciences, University of Coimbra, Calçada Martim de Freitas, 3000-456 Coimbra, Portugal; 3 Secció de Bioquímica i Biologia Molecular, Departament de Bioquímica i Fisiologia, Facultat de Farmàcia i Ciències de l’Alimentació, Universitat de Barcelona (UB), Joan XXIII 27, 08028 Barcelona, Spain

Correction to: *Scientific Reports* 10.1038/s41598-017-19087-y, published online 11 January 2018

The Acknowledgements section in this Article is incomplete.

“Structural funding for the Center for Neuroscience and Cell Biology and Centre for Functional Ecology was provided by FCT/MEC (Portugal) through national funds and the co-funding by the FEDER, within the PT2020 Partnership Agreement, and COMPETE 2020, within the projects POCI-01-0145-FEDER-007440 and UID/BIA/04004/2013, respectively. Authors also acknowledge FCT for funding in the form of fellowships to João Rito (SFRH/BD/87056/2012) and Ivan Viegas (SFRH/BPD/90032/2012). The authors also acknowledge financial support in the form of research grants from MEC (Spain) (AGL2012-33305 and AGL2016-78124-R; co-funded by the ERDF). NMR data was collected at the UC-NMR facility that is supported by FEDER and FCT (RECI/ QEQ-QFI/0168/2012, CENTRO-07-CT62-FEDER-002012), and Rede Nacional de Ressonância Magnética Nuclear (RNRMN). European seabass for the experiments were kindly provided by Eng. Canas.”

should read:

“Structural funding for the Center for Neuroscience and Cell Biology (CNC) and Centre for Functional Ecology (CFE) was provided by FCT/MEC (Portugal) through national funds and the co-funding by the FEDER, within the PT2020 Partnership Agreement, and COMPETE 2020, within the projects POCI-01-0145-FEDER-007440 and POCI-01-0145-FEDER-016828 (PTDC/CVT-NUT/2851/2014) from CNC and UID/BIA/04004/2013 from CFE. Authors also acknowledge FCT for funding in the form of fellowships to João Rito (SFRH/BD/87056/2012) and Ivan Viegas (SFRH/BPD/90032/2012). The authors also acknowledge financial support in the form of research grants from MEC (Spain) (AGL2012-33305 and AGL2016-78124-R; co-funded by the ERDF). NMR data was collected at the UC-NMR facility that is supported by FEDER and FCT (RECI/ QEQ-QFI/0168/2012, CENTRO-07-CT62-FEDER-002012), and Rede Nacional de Ressonância Magnética Nuclear (RNRMN). European seabass for the experiments were kindly provided by Eng. Canas.”

